# Spatiotemporal trends of HIV among international migrants: a 20-year surveillance study at Sichuan Ports, China

**DOI:** 10.3389/fpubh.2025.1681756

**Published:** 2025-12-16

**Authors:** Ying Shi, Qing Zhang, Lvbo Tian, Xuelong Chen, Juan Long

**Affiliations:** 1 Sichuan International Travel Healthcare Center (Chengdu Customs Port Clinic), Sichuan International Travel Healthcare Center (Chengdu Customs Port Outpatient Department), Chengdu, Sichuan, China; 2 Department of Transfusion Medicine, Sichuan Academy of Medical Sciences and Sichuan Provincial People's Hospital, School of Medicine, University of Electronic Science and Technology of China, Chengdu, Sichuan, China; 3 Department of Laboratory Medicine and Sichuan Provincial Key Laboratory for Human Disease Gene Study, Sichuan Provincial People's Hospital, School of Medicine, University of Electronic Science and Technology of China, Chengdu, Sichuan, China

**Keywords:** HIV, AIDS, epidemiological characteristics, prevention, ports

## Abstract

**Objective:**

To investigate HIV epidemiological characteristics and risk factors among international migrants (including Chinese citizens departing/returning and foreign nationals entering China) at Sichuan International Travel Health Care Center (2004–2024), informing targeted prevention strategies.

**Methods:**

A retrospective analysis of HIV infections was conducted using ELISA screening and Western blot confirmation.

**Results:**

Out of 566,475 individuals screened, 323 were found to be positive, resulting in a positivity rate of 0.057% (95% CI: 0.049–0.065). The characteristics of the infected individuals were as follows: (1) the majority were male (92.57%), predominantly aged between 41 and 50 years (29.33%), and primarily comprised migrant workers (59.49%); (2) the CRF55_01B subtype was the most prevalent, accounting for 60.61%, and exhibited an independent transmission chain; (3) heterosexual transmission was the primary mode of infection (47.68%), with 74.37% of individuals not maintaining consistent condom use. Additionally, there has been an increasing trend in the proportion of individuals aged 51–60 and those with a college education or higher in recent years.

**Conclusions:**

A comprehensive review of two decades of data indicates the necessity for port-focused interventions aimed at middle-aged migrant workers, improved surveillance measures, and the incorporation of molecular epidemiology into prevention strategies. Interventions should be specifically tailored to prioritize evidence-based approaches for high-risk groups, particularly mobile workers aged 51–60 years and university students engaging in unprotected sexual activities.

## 1 Background

Human immunodeficiency virus/acquired immune deficiency syndrome (HIV/AIDS) remains a significant global health challenge (1), despite extensive research and public health initiatives over the years. The high mutation rate of HIV (2), coupled with the lack of a cure or preventive vaccine (3), poses a considerable threat to human health. According to the latest epidemiological data from the Global Burden of Disease (GBD) study in 2019 (4), HIV/AIDS continues to be one of the leading causes of death among young adults worldwide (5), with sub-Saharan Africa experiencing the highest burden of new infections and AIDS-related mortality (6).

The epidemiology of HIV/AIDS is multifaceted, influenced by factors such as population mobility, sexual behavior, and substance use, which collectively shape the dynamics of virus transmission (7). Effective surveillance systems are critical for monitoring the epidemic, guiding public health interventions, and evaluating their effectiveness. However, HIV infection surveillance faces numerous challenges, necessitating not only accurate and timely data collection but also cultural sensitivity (8). Additionally, changing patterns of HIV/AIDS, including increasing prevalence among men who have sex with men and young women, require surveillance systems to be flexible and adaptable (9).

Sichuan Province serves as a critical transportation and economic hub in Western China, connecting the country to Central Asia, Southeast Asia, and Europe through the Belt and Road Initiative (BRI) (10). Key infrastructure includes the Chengdu Shuangliu International Airport and Chengdu Railway Port, which are vital nodes in China's international logistics network (11, 12). According to recent customs statistics, Chengdu Shuangliu International Airport ranked among the top five in mainland China for international passenger throughput in the post-pandemic period, handling approximately 8.5% of the nation's international air traffic. This underscores its significant role in national border health surveillance compared to other major ports like Beijing, Shanghai, and Guangzhou (13, 14). Therefore, effective monitoring of HIV among individuals entering and exiting the country (hereinafter referred to as international migrants, encompassing both Chinese and foreign nationals) through Sichuan ports, along with the application of the latest research and data, is vital for understanding the distribution and epidemiological characteristics of HIV. Such efforts will provide a robust foundation for developing targeted strategies and measures aimed at preventing and controlling HIV.

## 2 Materials and methods

### 2.1 Study design and data collection

From 2004 to 2024, a total of 566,475 individuals who entered and exited the country underwent HIV antibody testing at the Sichuan International Travel Health Care Center. Epidemiological studies of HIV-positive individuals were carried out using retrospective survey research methodologies. All data is anonymous without any health interventions. Each individual undergoing HIV testing was assigned a unique identifier. This identifier was used to seamlessly link the corresponding blood sample, laboratory test results, and questionnaire data within the center's information management system. All data were stored securely in China CDC Infectious Disease Surveillance Reporting System, which has been maintained since 2004. Following data extraction, a rigorous cleaning process was implemented, including checks for logical errors and inconsistencies between different data sources; any identified discrepancies were verified against the original paper records by two research assistants independently.

The participants in this study cohort reflect only those who were monitored, rather than the entire population passing through the port. Testing is mainly focused on particular groups, including but not limited to: individuals seeking mandatory health certificates for international employment or education, those undergoing health evaluations for visa applications, and key populations who have resided abroad for over a year (such as long-term expatriates and frequent travelers)—these groups are strongly encouraged to participate in testing.

### 2.2 Antibody screening

Blood samples were obtained through vacuum venipuncture from individuals entering and leaving the country at the Sichuan International Travel Health Care Center for HIV antibody testing. The initial screening for HIV antibodies was performed using the enzyme-linked immunosorbent assay (ELISA; Livzon Diagnostic, China) in accordance with the manufacturer's instructions. Individuals who tested positive for HIV antibodies via ELISA were subsequently retested using the colloidal selenium assay (Alere, Inc, China). All laboratory procedures, including ELISA, colloidal selenium assay, and Western Blot, were conducted in a Biosafety Level 2 (BSL-2) facility at the Sichuan International Travel Health Care Center, adhering to standard operating protocols for handling potential pathogenic specimens.

### 2.3 Western blot confirmation

Reactive samples for the primary screening of HIV antibodies are validated using the HIV-1/2 Western Blot kit (Bio-Rad, USA). HIV-1 viral antigens are isolated through electrophoresis and subsequently electrotransferred onto membranes. Synthetic peptides specific to HIV-2 are then introduced to the membrane strips, facilitating the simultaneous detection of HIV-2 antibodies. Diluted serum or plasma samples, along with controls, are applied to each nitrocellulose reagent membrane strip and incubated. If the sample contains antibodies specific to HIV-1 and HIV-2, these antibodies will bind to the HIV-1 protein and HIV-2 peptide present on the reagent membrane. Unbound materials are eliminated through washing, allowing the specifically bound antibodies to attach to goat anti-human IgG antibodies conjugated with alkaline phosphatase. The presence of these antibodies can be visualized by adding a BCIP/NBT substrate, enabling the detection of trace amounts of serum or plasma. The assay is capable of identifying minimal quantities of HIV-specific antibodies in serum or plasma. The integrity of the reagent is confirmed through the inclusion of an internal control strip.

### 2.4 Genotyping procedures

Anticoagulated whole blood samples were collected and HIV-1 viral RNA was extracted from plasma using a nucleic acid extraction kit (Qiagen, Germany). The full-length HIV-1 genome was amplified by RT-PCR (SuperScript™ III One-Step RT-PCR System, Thermo Fisher, USA) with primers covering the major structural regions of the genome (gag, pol, env). The amplification products were purified and then subjected to high-throughput sequencing using the Illumina MiSeq platform (Illumina, USA). After obtaining the raw sequencing data, MAFFT software was used for sequence comparison and the best evolutionary model was determined by jModelTest. Finally, PhyML was utilized to construct a phylogenetic tree and reference sequences from the Los Alamos HIV database were used for genotyping.

### 2.5 Questionnaire

Data were collected via a WHO-adapted questionnaire, piloted for cultural validity (Cronbach's α = 0.85), and administered by trained staff to minimize recall bias. The questionnaire encompassed three key dimensions: (1) fundamental demographic characteristics (gender, age, occupation); (2) broader social determinants (marital status, education level, spousal status); and (3) behavioral risk factors (history of drug use, sexual contact, and blood product utilization). The design of the questionnaire adhered to the World Health Organization's guidelines for biobehavioral surveillance surveys and was culturally adapted and pilot-tested to ensure its local relevance. All responses were collected by trained personnel to reduce the potential for self-report bias.

### 2.6 Statistical analysis

Statistical analysis was performed using SPSS version 21.0. Measurement data were assessed for normal distribution and expressed as mean ± standard deviation (X ± S). Categorical data were reported as cases (%). Independent samples *t*-tests were employed to compare differences between groups, while χ^2^ tests were utilized to evaluate the abnormal rates of indicators across groups. A *P*-value of less than 0.05 was considered statistically significant.

## 3 Results

### 3.1 HIV/AIDS surveillance profile and incidence rate

Between 2004 and 2014, a dynamic surveillance study on HIV infection was carried out among the entry and exit populations at the ports of Sichuan Province. The fundamental characteristics of the monitored populations were shown in Table 1.

**Table 1:** Demographic characteristics of screened individuals and HIV-positive cases.

**Characteristic**	**Screened population (*n* = 566,475)**	**HIV-positive cases (*n* = 323)**
**Gender**, ***n*** **(%)**		
Male	312,568 (55.2%)	299 (92.6%)
Female	253,907 (44.8%)	24 (7.4%)
**Age**, ***n*** **(%)**		
≤ 20 years	38,518 (6.8%)	9 (2.8%)
21–30 years	198,266 (35.0%)	76 (23.6%)
31–40 years	164,277 (29.0%)	86 (26.7%)
41–50 years	113,691 (20.1%)	108 (33.3%)
>50 years	51,723 (9.1%)	44 (13.6%)
**Occupation**, ***n*** **(%)**		
Migrant workers	297,892 (52.6%)	192 (59.5%)
International students	76,118 (13.4%)	44 (13.7%)
Business professionals	85,294 (15.1%)	16 (5.0%)
Teacher	24,695 (4.3%)	29 (8.9%)
Others	82,476 (14.6%)	42 (12.9%)
**Marital status**, ***n*** **(%)**		
Married	335,618 (59.2%)	193 (59.9%)
Unmarried	230,857 (40.8%)	130 (40.1%)

**Figure 1:**
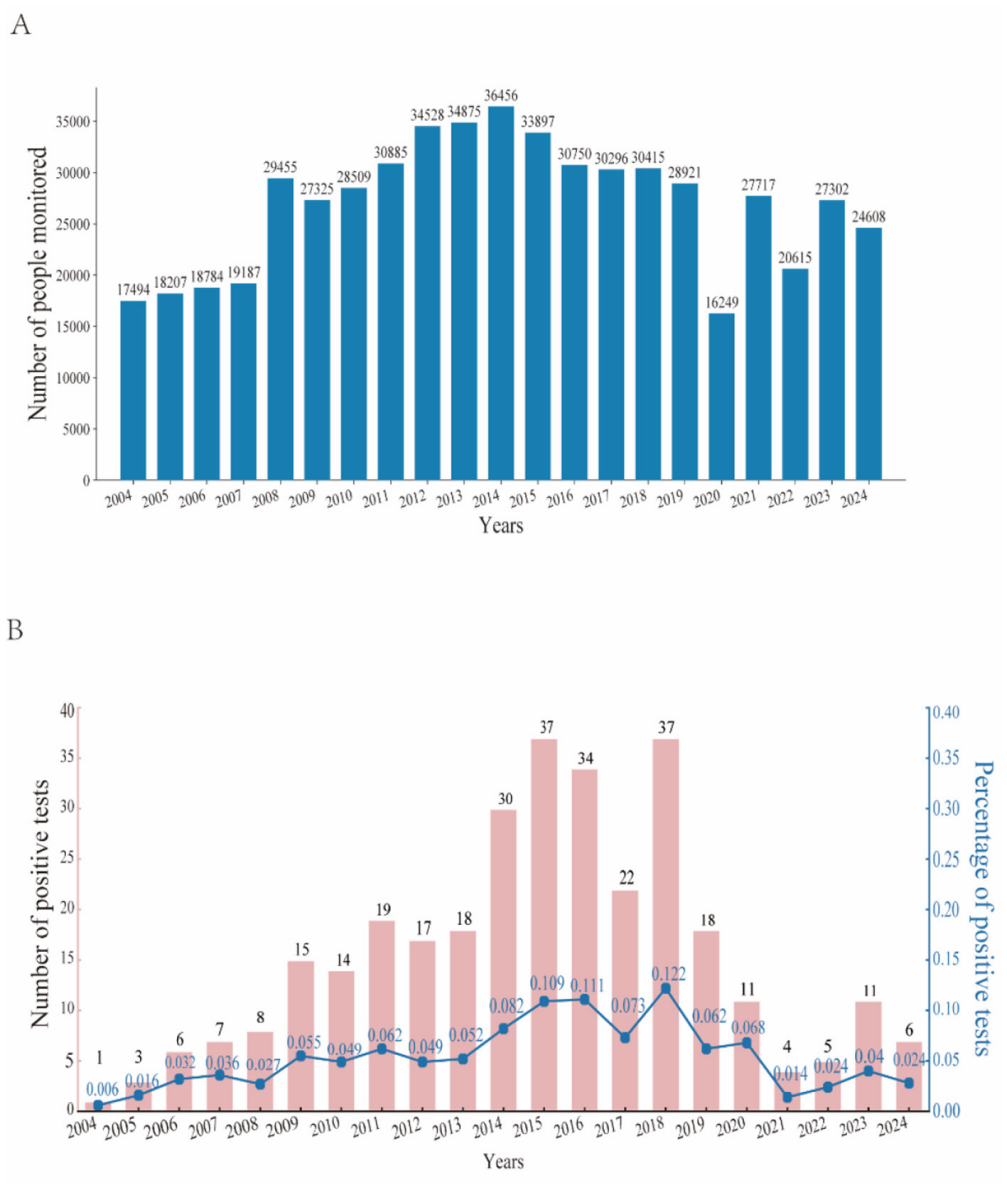
HIV/AIDS surveillance profile. **(A)** The annual number of individuals monitored from 2004 to 2024. **(B)** The number of positive detections and the positive detection rate from 2004 to 2024 are illustrated. The red bars indicate the number of positive detections, while the blue line represents the positive detection rate.

Surveillance data indicated (Figure 1A) that HIV monitoring in the ports of Sichuan Province exhibited significant fluctuations over time. The scale of surveillance expanded consistently from 2004 to 2014, peaking at 36,456 in 2014. However, following this peak, the number of surveillance cases declined annually, reaching 24,608 in 2024, which represents a 32.5% decrease from the peak year. A total of 323 positive tests were recorded, resulting in a positive detection rate of 0.057%. This detection rate displayed a double peak in 2015 (37 cases) and 2018 (37 cases; Figure 1B, pink bar graph). The highest positive detection rate (Figure 1B, blue dashed line) was observed in 2018 (0.122%), after which there has been a continuous decline, with the rate dropping to a historical low of 0.024% in 2024. It is noteworthy that the number of positive cases in 2018 was identical to that in 2015 (both 37 cases); however, due to a reduction in the number of individuals under surveillance (33,897 in 2015 compared to 30,415 in 2018), the positivity rate increased by 11.9% from 2015 (0.109 vs. 0.122%).

### 3.2 Epidemiological characteristics

#### 3.2.1 Gender and age distribution

**Figure 2:**
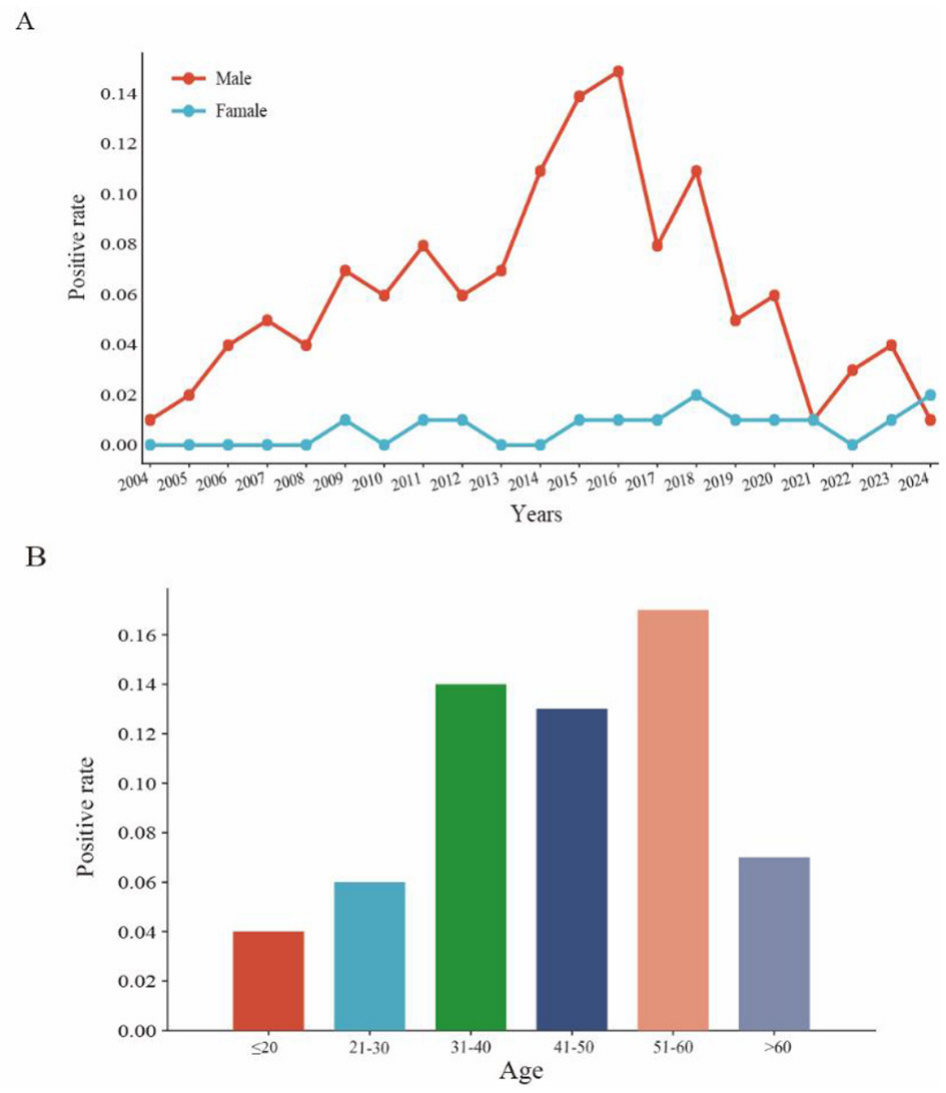
Gender and age distribution characteristics. **(A)** The solid red line represents males and the solid blue line represents females. **(B)** Positive rate stratified by age group.

Between 2004 and 2024, a notable gender disparity was observed in the HIV-positive detection rates at the ports of Sichuan Province, with males exhibiting a significantly higher positivity rate than females (χ^2^ = 41.07, *P* < 0.05; Figure 2A). The positivity rate among males demonstrated considerable fluctuations, following a pattern of “rapid increase-decrease-recovery”: it rose from 0.01% in 2004 to a peak of 0.15% in 2016, subsequently declining each year to 0.01% in 2021, before rebounding to 0.04% in 2023. In contrast, the female positivity rate remained relatively stable over time, fluctuating between 0.00 and 0.02%. The male positivity rate exhibited fluctuations that were 3.5 times greater than those of the female group, and the male-to-female ratio reached as high as 15:1 in 2016 (with male extremes at 0.15% compared to female extremes at 0.01%). This indicates that the male population consistently represents the primary risk group for HIV infection. The observed rebound in the male positivity rate from 2021 to 2023 may suggest the emergence of new transmission risk factors, which warrants further investigation in conjunction with epidemiological data.

Among the 323 HIV-positive cases identified from 2004 to 2024, ages varied from 15 to 68 years. The highest incidence was observed in the 41–50 age group, accounting for 33.33% of cases, followed by the 31–40 age group at 26.67%. Notably, between 2014 and 2021, the percentage of cases in the 51–60 age group rose significantly, increasing from 3.3% in 2014 to 50.0% in 2021 (Supplementary Table S1). The positivity rate for individuals aged 51–60 was 0.17%, which was significantly elevated than that of other age groups (χ^2^ = 18.37, *P* < 0.001; Figure 2B).

#### 3.2.2 Occupational and regional distribution

**Figure 3:**
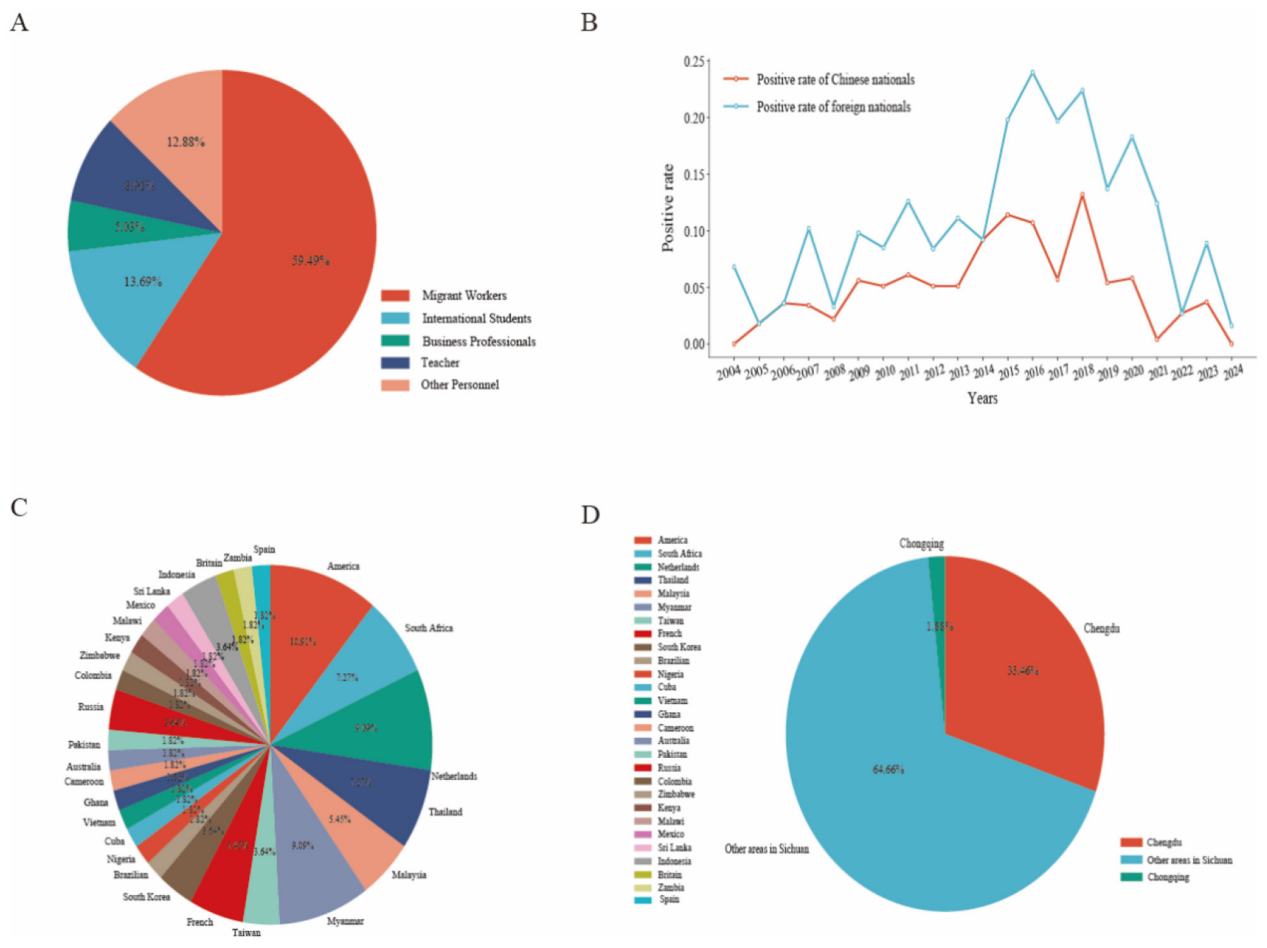
Occupational and regional distribution characteristics. **(A)** Occupational distribution of positive detections from 2004 to 2024. **(B)** Annual positive detection rates among Chinese nationals and foreign nationals from 2004 to 2024. **(C, D)** Composition of foreign **(C)** and Chinese **(D)** positive population.

Migrant workers represent the largest segment of the 323 reported HIV-infected cases, accounting for 59.49%. This proportion significantly surpasses that of other groups, including international students at 13.69%, business professionals at 5.03%, teachers at 8.91%, and others at 12.88% (Figure 3A).

Of the 323 HIV-infected cases, 266 (82.35%) were Chinese nationals returning from abroad, while 57 (17.65%) were foreign nationals entering China. The positivity rate among Chinese citizens increased from 0.00% to a peak of 0.132% between 2004 and 2018, followed by a gradual decline to 0.004% in 2021, before experiencing a resurgence in 2022–2023. In contrast, foreign nationals exhibited a higher positivity rate, which rose from 0.068% in 2004 to 0.232% in 2016, subsequently decreasing to 0.016% by 2024 (Figure 3B). Among the infected foreign nationals, the leading countries of origin included the United States (10.91%), South Africa (7.27%), the Netherlands (9.09%), Thailand (7.27%), and Malaysia (5.45%), collectively accounting for over 40% of the reported infections (Figure 3C). Of the 266 AIDS cases documented in China, 261 were from Sichuan Province, while only five cases were reported from Chongqing Municipality. Within Sichuan Province, Chengdu had the highest incidence, reporting 89 cases, which represented 33.46% of the total, significantly surpassing other regions in the province (Figure 3D).

#### 3.2.3 Molecular typing

**Figure 4:**
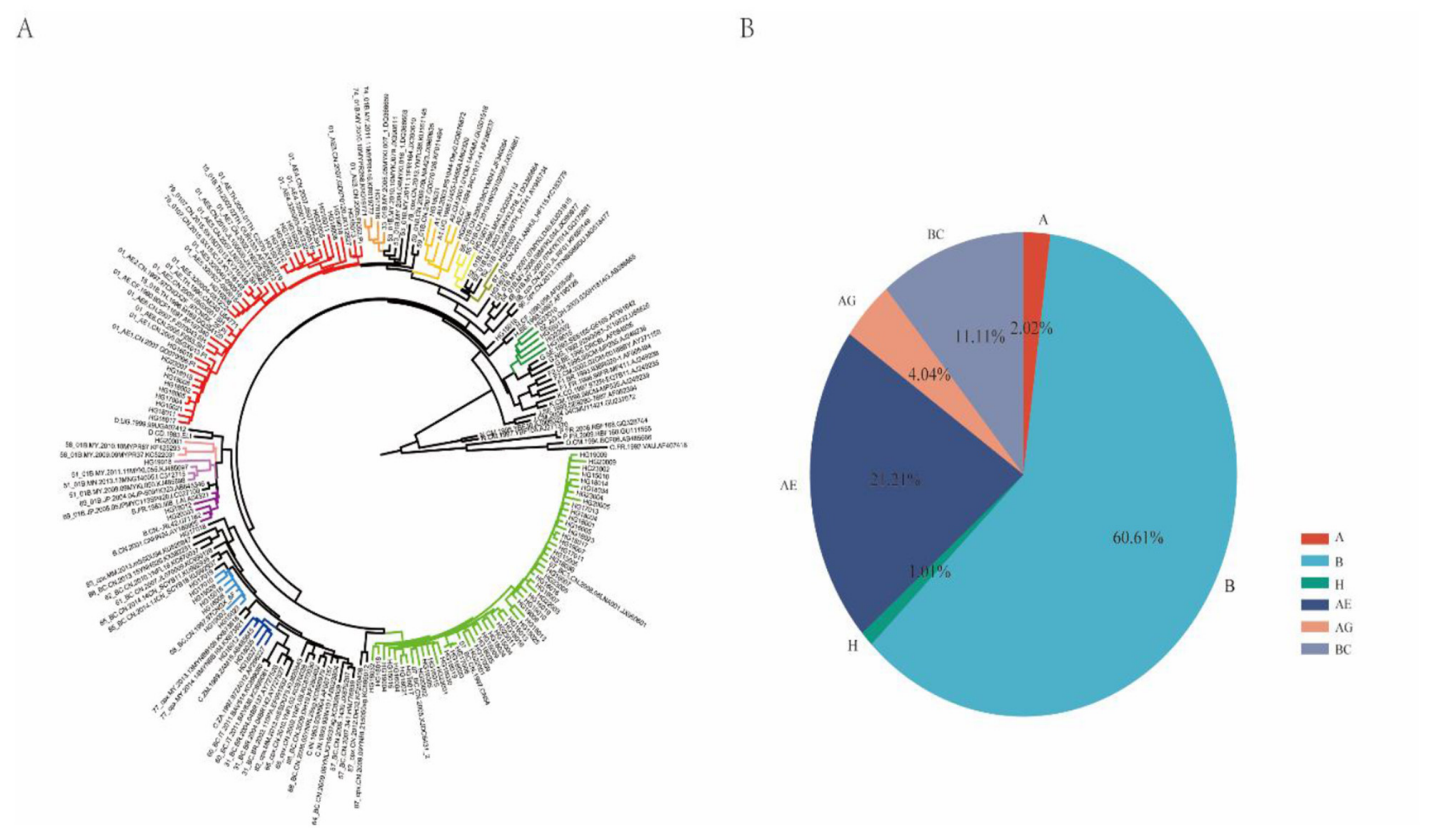
Molecular typing characteristics. **(A)** Construction and analysis of sequencing libraries and phylogenetic trees for positive samples. **(B)** Genotyping of positive samples.

Surveillance data indicated that all HIV strains identified at Sichuan ports were of the HIV-1 type. The predominant subtypes included CRF07_BC (BC), CRF01_AE (AE), CRF55_01B (B), CRF02_AG (AG), and subtype A. Phylogenetic analysis revealed a diverse array of subtypes and the presence of an independent transmission chain (Figure 4A). CRF55_01B (B) was the most prevalent, accounting for 60.61% of the population, followed by CRF01_AE (AE) at 21.21%, CRF07_BC (BC) at 11.11%, CRF02_AG (AG) at 4.04%, subtype A at 2.02%, and subtype H at 1.01% (Figure 4B).

### 3.3 Transmission pathways and associated risk factors

#### 3.3.1 Educational background

**Figure 5:**
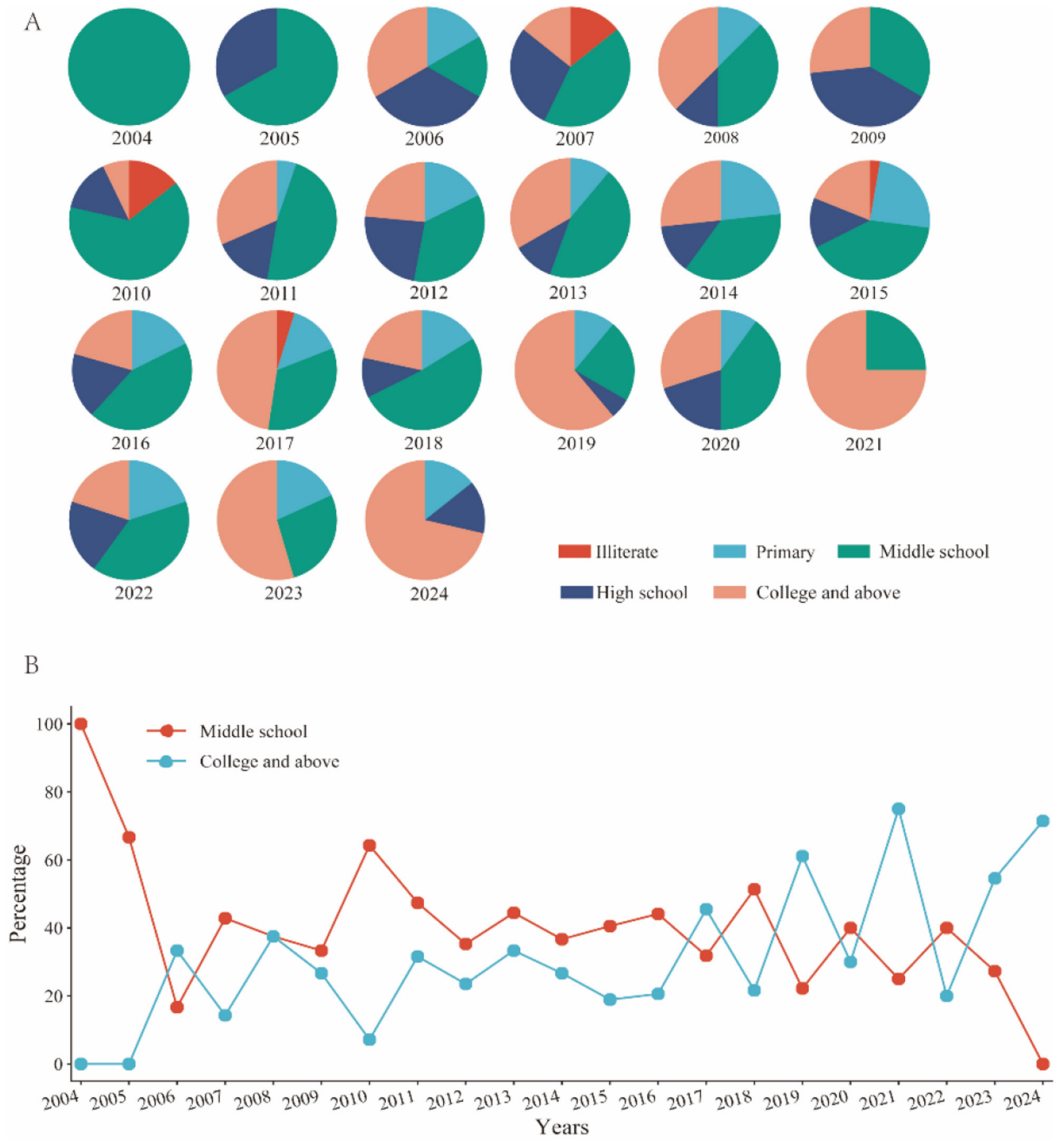
Educational distribution characteristics. **(A)** Educational distribution of infected individuals from 2004 to 2024. **(B)** The percentage of infected individuals with a middle school education and college and above from 2004 to 2024.

Between 2004 and 2024, a total of 323 HIV infection cases were reported, with the largest proportion of individuals having completed junior high school education, accounting for 39.94% (129 cases). This was followed by those with a college education or higher at 29.72% (96 cases), high school education at 14.55% (47 cases), primary education at 14.24% (46 cases), and illiterate individuals at 1.55% (five cases; Figure 5A). The percentage of individuals with tertiary education and above increased significantly from 0% in 2004 to 71.4% in 2024, particularly accelerating after 2017, although this growth exhibited considerable fluctuations. While the population with a junior high school education initially dominated, their representation decreased dramatically from 100% in 2004 to 0% in 2024 (Figure 5B).

#### 3.3.2 Marital status and transmission pathways

**Figure 6:**
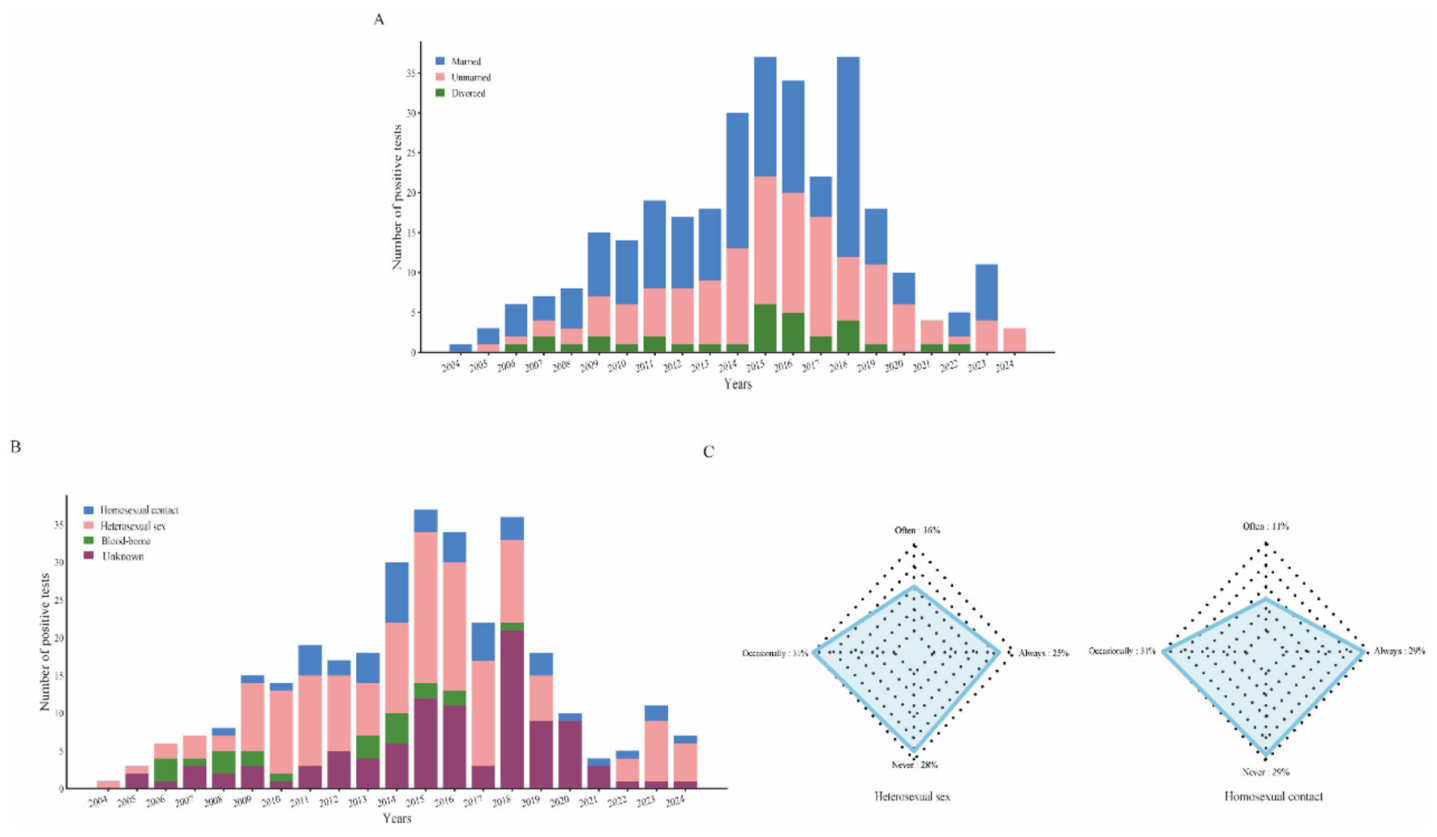
Marital status and transmission pathways. **(A)**The blue bar represents married, the pink bar represents unmarried, and the green bar represents divorced. **(B)** Distributional characteristics of transmission routes between 2004 and 2024. **(C)** Prevalence of condom use in heterosexual **(left)** and homosexual **(right)** encounters.

Between 2004 and 2024, among the 323 individuals diagnosed with HIV, 59.85% were married, reaching a high of 71.4% in 2015. Unmarried individuals constituted 40.15%, exhibiting notable fluctuations, with a peak of 68.2% in 2017. The proportion of divorced individuals was 9.91% (Figure 6A). Throughout the period, the percentage of married individuals consistently surpassed that of unmarried individuals, except for the years 2019 to 2021.

Between 2004 and 2024, a total of 323 HIV infections were recorded, with heterosexual contact identified as the predominant mode of transmission in 154 instances (47.68%). This was followed by homosexual contact, which accounted for 45 cases (13.93%). Blood transmission was reported in only 22 cases (6.81%), while the remaining cases were classified as unknown, comprising 31.58% (Figure 6B). The incidence of heterosexual transmission rose from 1 case (100% of cases) in 2004 to 20 cases (54.1% of cases) in 2015, after which there was a gradual decline in the number of positive cases. Among the 199 cases of sexually transmitted HIV infections, the reported patterns of condom use were as follows: 51 individuals indicated consistent use, 29 indicated essential use, 63 reported occasional use, and 56 reported never using condoms (Figure 6C). Statistical analysis (χ^2^ = 1.34, *P* > 0.05) indicated no significant difference in condom use between individuals infected through heterosexual vs. homosexual contact.

## 4 Discussion

Surveillance data from Sichuan ports spanning the years 2004 to 2024 offer crucial insights into the epidemiology of HIV in this highly mobile region. In the last 20 years, the average detection rate of HIV-positive individuals at entry points in Sichuan has been 0.057%. This rate is consistent with the national detection rate in China (15–17); however, it is lower than that observed in other developing nations (18–20). Prior to 2013, the detection rate at these ports remained below 0.06%, which was lower than the national HIV prevalence rate for that year. However, since 2014, there has been a significant increase in the number of detected HIV cases, resulting in a rapid escalation of detection rates. This surge is likely associated with China's emphasis on promoting the BRI initiative during the Boao Forum for Asia in 2014 (21), which has led to a notable rise in labor exports, with Sichuan being a key contributor to this workforce. Comparing our findings with port surveillance data from other major gateway cities in China holds significant value for understanding regional disparities (22–24), while also providing key insights into the epidemiological characteristics of the mobile population connecting Sichuan to international networks. Surveillance data indicate that the majority of individuals living with HIV are male, aged between 41 and 50, primarily migrant laborers with a junior high school education. The most prevalent strain identified is CRF55_01B, with heterosexual sexual contact being the primary mode of transmission. Recent epidemiological trends reveal several noteworthy developments: (1) over the past decade, there has been an increase in the number of individuals aged 50 and older who are HIV-positive, with this age group exhibiting a higher positivity rate compared to others; (2) the HIV positivity rate among foreign nationals surpasses that of Chinese citizens, with high-risk sources identified as the United States, South Africa, the Netherlands, Thailand, and Malaysia, highlighting the necessity for enhanced surveillance of individuals entering from these regions; (3) there has been a notable rise in the number of HIV-infected individuals aged 50 and above, particularly after 2017, alongside an accelerated increase in the proportion of those with a college education or higher.

The distribution of HIV-infected individuals in Sichuan ports is notably uneven, particularly in the peripheral regions surrounding Chengdu. This disparity is influenced by various factors, including population size, economic conditions, and population mobility (25). The proximity of these areas to Chengdu, coupled with well-developed transportation networks, facilitates high levels of population movement and access to medical services, thereby enhancing surveillance coverage (26). Furthermore, the concentration of colleges and universities in the Chengdu area has contributed to a significant annual increase in monitored HIV infections among international students (13). The age distribution of the infected population is also uneven, with individuals aged 41–50 years representing 33.3% of the cases. Additionally, there has been a notable rise in the number of infections among those aged 50 and older in recent years, which contrasts with the global trend of higher HIV incidence among younger populations (27). This divergence may indicate delayed diagnoses in older adults or specific risk behaviors associated with cross-border contexts.

In this study, 199 cases (61.6%) were attributed to sexual transmission, comprising 45 cases of same-sex contact and 154 cases of heterosexual contact. A significant proportion of those infected were expatriate laborers, a highly mobile demographic that, along with prolonged periods without stable sexual partners, heightens the likelihood of engaging in commercial sex and other high-risk sexual activities (28). Among those infected through heterosexual contact, 43 individuals (27.9%) reported never using protection, while only 39 (25.3%) consistently used condoms. The resurgence of heterosexual transmission (47.68%) as the predominant mode of transmission, combined with inadequate condom usage (74.7%), underscores the insufficiency of current prevention strategies for transnational laborers. Furthermore, the risk of HIV transmission through unprotected sex is intensified by the limited education of these groups and their lack of awareness regarding AIDS and its prevention, influenced by various socio-economic factors, including occupational hazards and healthcare access. Therefore, it is crucial to enhance HIV surveillance among foreign workers and their spouses, with a focus on controlling infection sources and interrupting transmission pathways, as well as to implement regular health education initiatives to improve awareness of the disease.

In recent years, there has been a significant rise in the number of highly educated individuals diagnosed with HIV at Sichuan ports, suggesting that HIV is beginning to infiltrate colleges and universities. This trend highlights substantial gaps in the preventive education regarding HIV within these institutions. The advent of Internet-based culture has led to an increase in high-risk sexual behaviors among college students, with bisexual individuals potentially serving as a “bridge” population that facilitates the transmission of HIV from high-risk homosexual groups to the wider heterosexual community (29). Although students have access to HIV-related information through various media outlets (30), the combination of instinctual excitement, physiological urges, and the absence of formal educational resources necessitates that colleges and universities enhance their HIV health education programs. A systematic, comprehensive, and in-depth approach is essential to effectively curb the spread of HIV on campus.

While our research offered longitudinal surveillance data, this retrospective study conducted at a single center has notable limitations: (1) limited data coverage from one center, leading to potential systematic bias; (2) concerns regarding questionnaire reliability, as responses may be influenced by individual subjectivity and recall bias; and (3) challenges in sampling the mobile population, characterized by high mobility within port areas and a low rate of proactive detection, which impacts the representativeness of the monitoring efforts.

## 5 Conclusion

Sichuan Province, which has a high rate of HIV infections (31), has enacted the “Four Free and One Care Policy” for individuals living with HIV and has implemented various measures to prevent and control AIDS. These measures include health education, targeted interventions for high-risk groups, and community-based comprehensive strategies. Nevertheless, the ongoing complexities of population mobility, cultural influences, and social dynamics have contributed to a continued rise in HIV infections. The situation remains critical, with a substantial gap between the current circumstances and the United Nations' 2016 objective of “ending the AIDS epidemic by 2030,” which seeks to achieve zero new HIV infections annually (32). In response to this challenge, the present study examines the epidemiological distribution and trends of the AIDS epidemic over the past two decades utilizing port data. While this 20-year surveillance has provided valuable insights into the epidemiology of HIV among migrant populations, data from single-center studies are limited and should be interpreted in conjunction with national epidemiology data. However, this study examines the epidemiological distribution characteristics and changing trends of the AIDS epidemic over the past 20 years using data from Sichuan ports. This analysis can still contribute to the surveillance and early warning of AIDS in Sichuan, thereby enhancing the proactivity, foresight, and timeliness of AIDS prevention and control efforts.

## Data Availability

The original contributions presented in the study are included in the article/Supplementary material, further inquiries can be directed to the corresponding authors.
