# Supplementary material for: Spatiotemporal trends of HIV among international migrants: a 20-year surveillance study at Sichuan Ports, China
Source: Front Public Health. 2025 Dec 16;13:1681756. doi: 10.3389/fpubh.2025.1681756 (PMC12748186; doi:10.3389/fpubh.2025.1681756)
Supplement: Supplementary file 1 [file Table_1.docx]

**Supplementary table 1. Age demographics of HIV-positive individuals, 2004–2024**

| Year | ≤20 | 21～30 | 31～40 | 41～50 | 51～60 | ＞60 |
| --- | --- | --- | --- | --- | --- | --- |
| 2004 | 0 | 0 | 1 | 0 | 0 | 0 |
| 2005 | 0 | 0 | 2 | 1 | 0 | 0 |
| 2006 | 1 | 1 | 1 | 3 | 0 | 0 |
| 2007 | 0 | 1 | 3 | 2 | 1 | 0 |
| 2008 | 0 | 1 | 4 | 1 | 2 | 0 |
| 2009 | 1 | 2 | 6 | 2 | 3 | 1 |
| 2010 | 0 | 4 | 3 | 6 | 1 | 0 |
| 2011 | 0 | 4 | 7 | 7 | 0 | 1 |
| 2012 | 1 | 3 | 5 | 4 | 3 | 1 |
| 2013 | 2 | 4 | 6 | 6 | 0 | 0 |
| 2014 | 2 | 8 | 7 | 12 | 1 | 0 |
| 2015 | 0 | 14 | 4 | 13 | 6 | 0 |
| 2016 | 0 | 10 | 10 | 10 | 4 | 0 |
| 2017 | 1 | 5 | 5 | 7 | 3 | 1 |
| 2018 | 2 | 6 | 8 | 17 | 4 | 0 |
| 2019 | 2 | 8 | 2 | 4 | 2 | 0 |
| 2020 | 1 | 3 | 2 | 1 | 3 | 0 |
| 2021 | 0 | 0 | 1 | 1 | 2 | 0 |
| 2022 | 0 | 1 | 1 | 3 | 0 | 0 |
| 2023 | 0 | 1 | 4 | 4 | 2 | 0 |
| 2024 | 1 | 2 | 2 | 1 | 1 | 0 |
| Total | 14 | 70 | 84 | 105 | 38 | 4 |
